# Improving the Identification, Assessment and Management of Osteoporosis and Fragility Fracture Risk on a Later Life Psychiatry Ward: A Complete Audit Cycle

**DOI:** 10.1192/bjo.2022.340

**Published:** 2022-06-20

**Authors:** Morwenna Senior, Thomas Hewson, Francesca Brownless, Ross Dunne

**Affiliations:** Greater Manchester Mental Health NHS Foundation Trust, Manchester, United Kingdom

## Abstract

**Aims:**

Osteoporosis is common amongst elderly patient populations and is associated with significant morbidity and mortality. We aimed to assess whether national clinical guidelines regarding the identification, assessment and management of osteoporosis and fragility fracture risk were being adhered to on a female later life psychiatry ward. We then aimed to improve the detection and treatment of osteoporosis amongst this patient cohort and subsequently conducted a re-audit of adherence to relevant clinical guidelines.

**Methods:**

In July 2021, the electronic health records of the 20 most recently discharged patients from a female later life psychiatry ward were reviewed. The proportion of patients who appropriately received FRAX screening, DEXA scanning and pharmacological management of osteoporosis and fragility fracture risk was recorded. The results were compared to standards identified in national clinical guidelines from the National Institute for Health and Care Excellence (NICE) and the National Osteoporosis Guideline Group (NOGG). In addition, the proportion of patients who had FRAX scores communicated to their general practitioners on discharge was recorded. Recommendations were made based on audit findings, and several changes to ward processes were implemented including incorporating fracture risk scoring in a structured ward round template and displaying information posters about osteoporosis in clinical areas. A re-audit was completed in February 2022 using the same methodology as baseline to re-assess adherence to the audit standards.

**Results:**

All included patients were female and aged >65 years, and therefore eligible for consideration of fragility fracture risk according to NICE guidelines. 88% (15/17 patients) of those without pre-existing osteoporosis had FRAX scores calculated during their admission on re-audit compared to 50% (8/16 patients) at baseline. 73% (11/15 patients) had FRAX scores communicated to their GP on discharge at completion of the audit cycle compared to 25% (2/8 patients) at baseline. At completion of the audit cycle 10% (1/10 patients) with intermediate fragility fracture risk received measurement of bone mineral density during admission while 30% (3/10) had this recommended to their GP on discharge. None of the high-risk patients (n = 4) were started on bisphosphonate therapy.

**Conclusion:**

On completion of the audit cycle, we found excellent compliance with national guidelines regarding the identification of osteoporosis and fragility fracture risk, which demonstrates the feasibility of considering this aspect of physical health in the setting of a later-life psychiatry ward. Areas for improvement include the assessment and management of patients identified as having intermediate or high risk of osteoporosis and fragility fractures.

